# Proteasome inhibition disrupts the metabolism of fumarate hydratase- deficient tumors by downregulating p62 and c-Myc

**DOI:** 10.1038/s41598-019-55003-2

**Published:** 2019-12-05

**Authors:** Carole Sourbier, Christopher J. Ricketts, Pei-Jyun Liao, Shingo Matsumoto, Darmood Wei, Martin Lang, Reema Railkar, Youfeng Yang, Ming-Hui Wei, Piyush Agarwal, Murali Krishna, James B. Mitchell, Jane B. Trepel, Len Neckers, W. Marston Linehan

**Affiliations:** 1 Urologic Oncology Branch, Center for Cancer Research, National Cancer Institute, Bethesda, Maryland United States of America; 2 Present Address: Office of Biotechnology Products, Office of Pharmaceutical Quality, Center for Drug Evaluation and Research, US Food & Drug Administration, Silver Spring, Maryland United States of America; 3 Radiation Biology Branch, Center for Cancer Research, National Cancer Institute, Bethesda, Maryland United States of America; 4 Division of Bioengineering and Bioinformatics, Graduate School of Information Science and Technology, Hokkaido University, Sapporo, Japan; 5 JST, PREST, Saitama, Japan; 6 Developmental Therapeutics Branch, Center for Cancer Research, NCI, Bethesda, Maryland United States of America

**Keywords:** Cancer metabolism, Renal cell carcinoma

## Abstract

Hereditary leiomyomatosis and renal cell carcinoma (HLRCC) is characterized by germline mutations of the *FH* gene that encodes for the TCA cycle enzyme, fumarate hydratase. HLRCC patients are at risk for the development of an aggressive form of type 2 papillary renal cell carcinoma. By studying the mechanism of action of marizomib, a proteasome inhibitor able to cross the blood-brain barrier, we found that it modulates the metabolism of HLRCC cells. Marizomib decreased glycolysis *in vitro* and *in vivo* by downregulating p62 and c-Myc. C-Myc downregulation decreased the expression of lactate dehydrogenase A, the enzyme catalyzing the conversion of pyruvate to lactate. In addition, proteasomal inhibition lowered the expression of the glutaminases *GLS* and *GLS2*, which support glutamine metabolism and the maintenance of the redox balance. Thus, in HLRCC cells, proteasome inhibition disrupts glucose and glutamine metabolism, restricting nutrients and lowering the cells’ anti-oxidant response capacity. Although the cytotoxicity induced by proteasome inhibitors is complex, the understanding of their metabolic effects in HLRCC may lead to the development of effective therapeutic strategies or to the development of markers of efficacy.

## Introduction

With an estimated 65,000 new cases and nearly 15,000 deaths in 2018, kidney cancer is the 12^th^ leading cause of death in the United States^1^. Renal cell carcinomas (RCC) have diverse histologies and can present in both a sporadic or inherited form. Much of what is known about the genetic basis of RCC has come from the study of the inherited forms of the disease, such as von Hippel-Lindau (VHL), hereditary papillary renal cell carcinoma (HPRC) and hereditary leiomyomatosis and renal cell carcinoma (HLRCC). The most prevalent type of RCC is clear cell RCC, representing about 75% of all RCC. Study of the VHL familial cancer syndrome led to the identification of the *VHL* tumor suppressor gene, which is also mutated or methylated in a high percentage of tumors from patients with sporadic clear cell RCC^2,3^. *VHL* encodes for the protein VHL which forms a complex with other proteins that play a major role in controlling the cells response to hypoxia^4,5^. The understanding of the molecular function of VHL provided the foundation for the development of targeted therapies against hypoxia-induced factors for patients with advanced clear cell RCC^4,6^. Papillary renal cell carcinoma (PRCC) accounts for about 15% of all RCC and is subcategorized into Type 1 and Type 2 PRCC. Studies of the familial form of Type 1 PRCC, HPRC, led to the identification of activating germline mutations in *MET*. This discovery led to the identification of mutations and amplification of *MET* in sporadic Type 1 PRCC^7,8^, and to the development of therapeutic approaches targeting the MET pathway in hereditary and sporadic PRCC. HLRCC is a hereditary cancer syndrome in which affected individuals are at risk for the development of cutaneous and uterine leiomyomas and an aggressive form of Type 2 PRCC^9,10^. It is characterized by a germline mutation of the gene for the TCA cycle enzyme fumarate hydratase (*FH*) and subsequent loss of the wild-type *FH* allele that results in complete inactivation of the fumarate hydratase enzyme (FH) in tumors^11^.

HLRCC-associated Type 2 PRCC has a distinctive histology with orangeophilic nucleoli and prominent perinucleolar halo. It presents with an aggressive clinical phenotype that has a propensity to metastasize early^10,12^. FH converts fumarate into malate; hence, loss of FH activity leads to a disruption of the TCA cycle and accumulation of intracellular fumarate. To survive, FH-deficient cells undergo a metabolic shift to aerobic glycolysis with impaired oxidative phosphorylation and a dependence upon glucose for survival^13–15^. Additionally, increased intracellular fumarate levels inhibit the prolyl hydroxylases responsible for hydroxylation of hypoxia inducible factor 1α (HIF1α), a necessary step for VHL-mediated degradation of HIF in normoxia^13,15–18^. This results in HIF1α stabilization which leads to the aberrant expression of HIF transcriptional target genes that promote glycolysis and angiogenesis^13,19^.

The metabolic shift of FH-deficient tumor cells to aerobic glycolysis also leads to increased reactive oxygen species (ROS) levels^15,20^. To survive an unbalanced redox homeostasis while still promoting growth and anabolic pathways, FH-deficient tumor cells depend on a strong antioxidant response. They enhance the NADPH production needed to produce glutathione via increased glucose uptake and shuttling of glucose-6-phosphate into the oxidative branch of the pentose phosphate pathway^21^. Additionally, fumarate accumulation results in succination of NRF2 inhibitor, KEAP1, leading to translocation of the NRF2 transcription factor from the cytoplasm to the nucleus resulting in activation of antioxidant response pathways^22,23^. NRF2 activation acts by promoting the expression of detoxifying proteins, such as NQO1 and HMOX1 to contain ROS below a level that would cause cellular damage. The establishment of HLRCC patient-derived renal cell line models that recapitulate the metabolic alterations observed in FH-deficient tumors has provided a valuable tool for delineating critical vulnerabilities in FH-deficient tumors^14,24–26^. We have previously shown that increasing ROS, by inhibiting the proteasomal function or by targeting the antioxidant response, were both effective preclinical approaches in FH-deficient cells^27,28^. The proteasome inhibitor, bortezomib, induced oxidative stress and was lethal to FH-deficient Type 2 PRCC cells *in vitro* and in patient-derived-xenograft (PDX) models, as a single agent or in combination with cisplatin that is also known to generate high ROS levels^27^.

HLRCC patients with renal tumors are at risk of metastatic disease as FH-deficient tumors have a propensity to metastasize early to a number of sites, including the lungs and brain. Brain metastases may be clinically challenging to treat as it is necessary for the systematic therapies to cross the blood-brain barrier (BBB). Despite the potent preclinical effects of bortezomib on FH-deficient cells, it has clinical limitations due to its inability to cross the BBB, while the second-generation proteasome inhibitor marizomib is BBB-permeant^29,30^. Thus, we investigated the antitumor effects of marizomib in FH-deficient nonclinical models.

## Results

### Marizomib is cytotoxic to *FH*-deficient tumor cells *in vitro* and induces tumor regression *in vivo* in a HLRCC xenograft animal model

**Figure 1:**
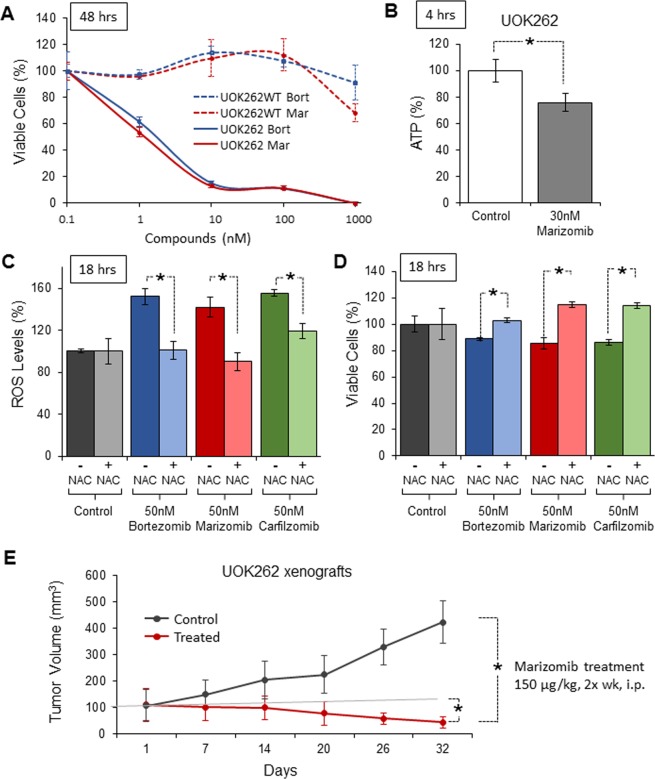
Marizomib is cytotoxic to HLRCC cells *in vitro* and *in vivo*. (**A**) UOK262 and UOK262WT cells were treated for 48 hours with either bortezomib (Bort) or marizomib (Mar). Cell viability was assessed by CellTiterGlo; (**B**) ATP content of UOK262 was evaluated 4 hours post-treatment with marizomib (Mar) using ATPLite assay; (**C**,**D**) UOK262 cells were treated with bortezomib, marizomib and carfilzomib. ROS levels were measured after 18 hours treatment using DCF dye (**C**) and viability was assessed in parallel using CellTiterGlo. The antioxidant NAC was used to counteract the build-up of ROS by pretreating the cells with 5 mM of NAC 4 hours prior to the addition of the proteasome inhibitors; (**E**) Tumor growth curve of a xenograft study with UOK262 injected in the flank of female nude mice and treated with marizomib or vehicle (see methods). Tumors were measured weekly using calipers and tumor volume was calculated as length × width^2^. *p < 0.05; Control: DMSO control.

Inhibition of the proteasome using bortezomib showed promising anti-tumor effect in a HLRCC animal model^27^. In the current study, we assessed whether the second-generation proteasome inhibitor marizomib might have a similar pharmacological efficacy. The HLRCC-derived FH-deficient cell line UOK262 and its fumarate hydratase (FH)-restored counterpart, UOK262WT, were treated with a concentration range of bortezomib or marizomib for 48 h. UOK262 cells, but not UOK262WT, were highly sensitive to both proteasome inhibitors with comparable IC50 (IC50~5–6 nM, Fig. 1A). The cytotoxicity of marizomib at 4 h, 24 h and 48 h in UOK262 is illustrated in Fig. S1. Marizomib treatment also significantly decreased the levels of ATP in UOK262 cells by approximately 20% (Fig. 1B). Proteasome inhibitors are known to induce oxidative stress and bortezomib’s cytotoxic effect in UOK262 was, at least partially, ROS-dependent^27^. Thus, ROS levels were measured following treatments with bortezomib, marizomib and an additional second-generation proteasome inhibitor carfilzomib. All three proteasome inhibitors significantly increased ROS levels. That effect was reversed by the ROS scavenger N-acetyl-cystein^31^ (NAC; Fig. 1C). To counteract the build-up of ROS, UOK262 cells were pretreated with 5 mM of NAC (diluted in water) for 4 h prior to the addition of the proteasome inhibitors. Concordantly, the three proteasome inhibitors mildly decreased cell viability and this effect was abrogated with the addition of the ROS scavenger NAC suggesting that, like bortezomib, a component of carfilzomib’s and marizomib’s cytotoxicity was ROS-dependent (Fig. 1D). Since marizomib mimicked bortezomib *in vitro*, its effect *in vivo* was evaluated using a mouse xenograft model of HLRCC. Athymic female mice bearing subcutaneously implanted UOK262 xenografts were treated with marizomib (150 µg/kg, twice a week, i.p.) or with vehicle for a month. In every animal treated with marizomib (n = 8), tumors demonstrated significant regression after 32 days (Fig. 1E) and over an extended period of time 80% of mice had complete regression. Thus, in this HLRCC nonclinical model and similarly to bortezomib, marizomib displayed a potent *in vivo* efficacy.

### Proteasome inhibitors modulate HLRCC cells metabolism *in vitro* and *in vivo*

**Figure 2:**
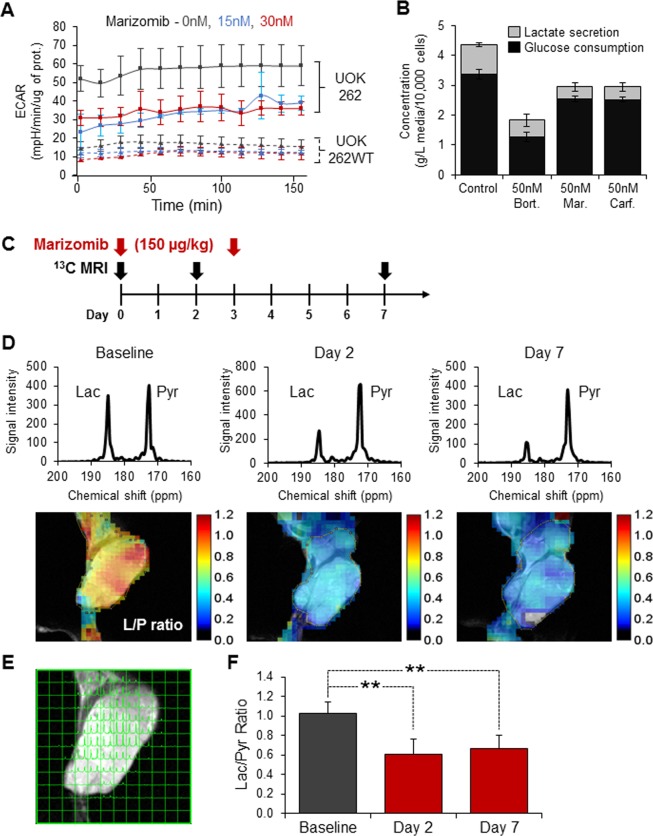
Marizomib inhibits aerobic glycolysis *in vitro* and *in vivo*. (**A**) Extracellular acidification rate (ECAR), a surrogate of lactate secretion, was measured using a seahorse technology platform following 4 hours treatment with marizomib; (**B**) Glucose and lactate levels were measured in the media of UOK262 cells 24 hours after treatment with bortezomib, marizomib or carfilzomib, using a biochemistry analyzer. C-F: *in vivo* metabolic effect of marizomib was assess by ^13^C-hyperpolarized pyruvate MRI imaging. The lactate (Lac) to pyruvate (Pyr) ratio was calculated at day 2 and day 7 post treatment (**C**,**D**). (**D**) Representative images of the lactate to pyruvate ratio from one animal; (**E**) Representative image with the signal intensity graph and the MRI imaging; **(F)** Average of the lactate to pyruvate ratio for 5 animals. **p < 0.01; Bort: bortezomib; Mar: marizomib; Carf: carfilzomib; Control: DMSO control; L/P: lactate to pyruvate ratio.

Since marizomib significantly affected UOK262’s ATP levels (Fig. 1B), its effect on the glycolytic pathway was evaluated. Extracellular acidification rate (ECAR), a surrogate of lactate secretion, was measured using the seahorse technology platform. UOK262 presented intrinsically higher rates of extracellular acidification compared to UOK262WT cells (Fig. 2A). Acute treatment with marizomib (15 nM or 30 nM, 4 h) significantly decreased ECAR in UOK262 cells while it did not affect UOK262WT cells (Fig. 2A). Analysis of the culture media from UOK262 cells collected 24 h post-treatment and normalized by cell number, confirmed this observation with all three proteasome inhibitors. UOK262 cells treated with bortezomib, marizomib and carfilzomib had reduced levels of both glucose consumption and lactate secretion (Fig. 2B). Together these data suggest that proteasome inhibition may decrease aerobic glycolysis in UOK262 cells. To assess whether this acute effect was reproducible *in vivo*, ^13^C-hyperpolarized pyruvate MRI imaging was performed on mice bearing UOK262 xenografts. Animals were imaged at day 0, day 2 and day 7; and were treated at day 0 and day 3 (Fig. 2C). The lactate to pyruvate ratio was significantly decreased at day 2 and day 7 indicating a reduced ability to convert pyruvate to lactate, a necessary step at the end of aerobic glycolysis (Fig. 2D–F). Thus, by inhibiting aerobic glycolysis in a HLRCC xenograft animal model, marizomib significantly modulates the metabolism of FH-deficient tumor cells *in vivo*.

### Proteasome inhibitors downregulate *LDHA* mRNA expression in a p62/c-Myc dependent manner

**Figure 3:**
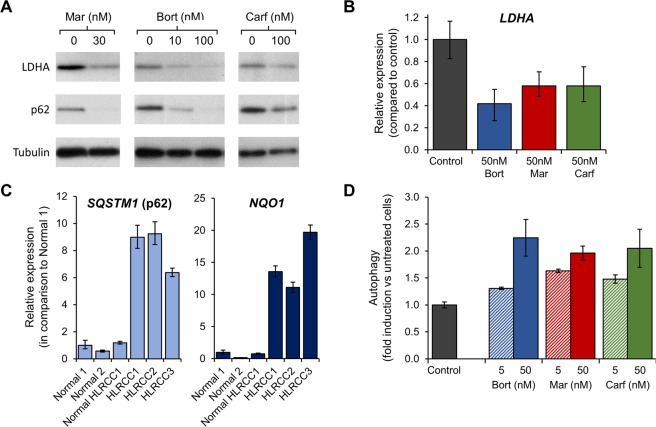
Proteasome inhibitors downregulate *LDHA* mRNA expression and induce autophagy. (**A**) LDHA and p62 protein expression in UOK262 cells were evaluated by immunoblotting 24 hours post-treatment with proteasome inhibitors. Tubulin was used as a loading control. Original, unprocessed images used are in Fig. S2; (**B**) *LDHA* mRNA expression was measured by RT-PCR 24 hours post-treatment; (**C**) Relative expression level of p62 (*SQSTM1*) and NQO1 (*NQO1*) by RT-PCR in HLRCC patients and healthy individuals. (**D**) Autophagy was measured in live cells by flow cytometry 24 hours post-treatment using a green fluorescent cationic amphiphilic tracer dye developed to stain phagophores, autophagosomes, and autolysosomes. *p < 0.05; Bort: bortezomib; Mar: marizomib; Carf: carfilzomib; Control: DMSO control.

How could proteasome inhibitors alter HLRCC cells metabolism? Lactate dehydrogenase (LDH) is the primary enzyme catalyzing the conversion of pyruvate into lactate. It is a tetramer composed of the LDHA and LDHB proteins encoded by the *LDHA* and *LDHB* genes, although the LDHA subunit is often predominant in highly glycolytic cells^32^. Treatment with all three proteasome inhibitors decreased both LDHA protein expression (Fig. 3A) and *LDHA* mRNA expression (Fig. 3B) in UOK262 cells, suggesting that the proteasome inhibitors altered *LDHA* transcription. A study from Valencia and collaborators showed that decreased p62 levels resulted in downregulation of c-Myc that alters glycolysis by decreasing c-Myc-induced transcription of *LDHA*^33^. Within HLRCC tumors, the NRF2 antioxidant response pathway is aberrantly upregulated and this results in increased expression of the NRF2 transcriptional targets such as *SQSTM1* (that encodes p62) and *NQO1* (Fig. 3C). A common effect of proteasome inhibition is the induction of autophagy^34^ that leads to p62 degradation. All three proteasome inhibitors induced autophagy in a dose-dependent manner (Fig. 3D), which resulted in loss of p62 protein expression in the UOK262 cells (Fig. 3A).

**Figure 4:**
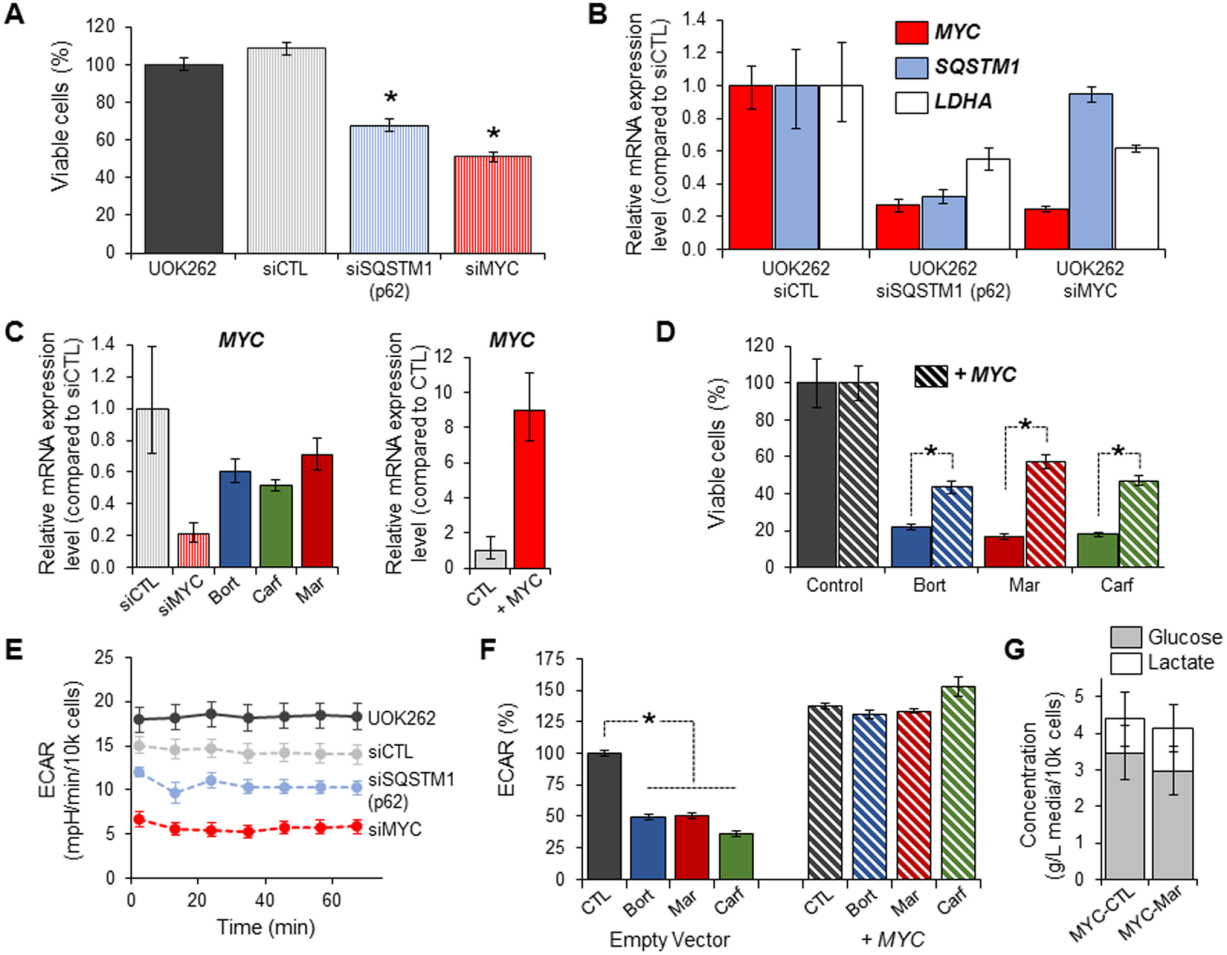
P62 and c-Myc mediate the metabolic effect of the proteasome inhibitors in UOK262 cells. (**A**) UOK262 cells were transiently silenced for p62 or c-Myc using pooled siRNA. Viability was assessed by luminescence 72 hours post-transfection; (**B**) Relative mRNA expression of *MYC*, *SQSTM1* (p62) and *LDHA* were measured by RT- PCR 48 hours after transfection with siRNA targeting p62 (*SQSTM1*) or *MYC*; (**C**) *MYC* expression levels were evaluated after 24 hours treatment with bortezomib, marizomib and carfilzomib (30 nM). Transient silencing of *MYC* was used as a control. Transfection of *MYC* expression plasmid in UOK262 cells was shown to result in significant overexpression of *MYC*; (**D**) *MYC* overexpression partially rescues the viability of UOK262 24 hours after bortezomib, marizomib and carfilzomib treatments (30 nM); (**E**) Transient silencing of p62 and c-Myc significantly decreased ECAR in UOK262 cells. Measurements were performed 48 hours after transfection; (**F**) Overexpression of *MYC* negates the effect of proteasome inhibitors on ECAR; (**G**) Overexpression of *MYC* abrogates the effect of marizomib on the lactate secretion and glucose consumption of UOK262 cells. *p < 0.05; Bort: bortezomib; Mar: marizomib; Carf: carfilzomib; Control: DMSO control; ECAR: extra-cellular acidification rate.

To investigate if p62 or c-Myc could play a role in the metabolic effect of the proteasome inhibitors, both p62 (*SQSTM1*) and c-Myc (*MYC*) were transiently silenced using pooled small interference RNA (siRNA). Silencing of either p62 (*SQSTM1*) or c-Myc (*MYC*) significantly, but not completely, decreased UOK262 cell viability (Fig. 4A). Silencing of *SQSTM1* expression resulted in downregulation of both *MYC* and *LDHA* mRNA expression, while silencing of *MYC* only reduced *LDHA* mRNA expression without altering *SQSTM1* expression (Fig. 4B). This supports the hypothesis that c-Myc regulation is downstream of p62 and *LDHA* expression regulation is downstream of both p62 and c-Myc. Furthermore, the three proteasome inhibitors significantly decreased *MYC* expression in UOK262 (Figs. 4C and S3) while UOK262 cells overexpressing *MYC* were partially resistant to the cytotoxic effect of the proteasome inhibitors (Fig. 4D). Finally, silencing of p62 and c-Myc both significantly reduced ECAR in UOK262 cells (Fig. 4E), while overexpression of *MYC* in UOK262 reversed the effect of the three proteasome inhibitors on ECAR (Fig. 4F) as well as on lactate secretion and glucose consumption (Fig. 4G). Taken together these data demonstrate that the metabolic effect of proteasome inhibition is at least partially mediated by the p62/c-Myc pathway.

### c-Myc modulation by the proteasome inhibitors affects glutamine metabolism

**Figure 5:**
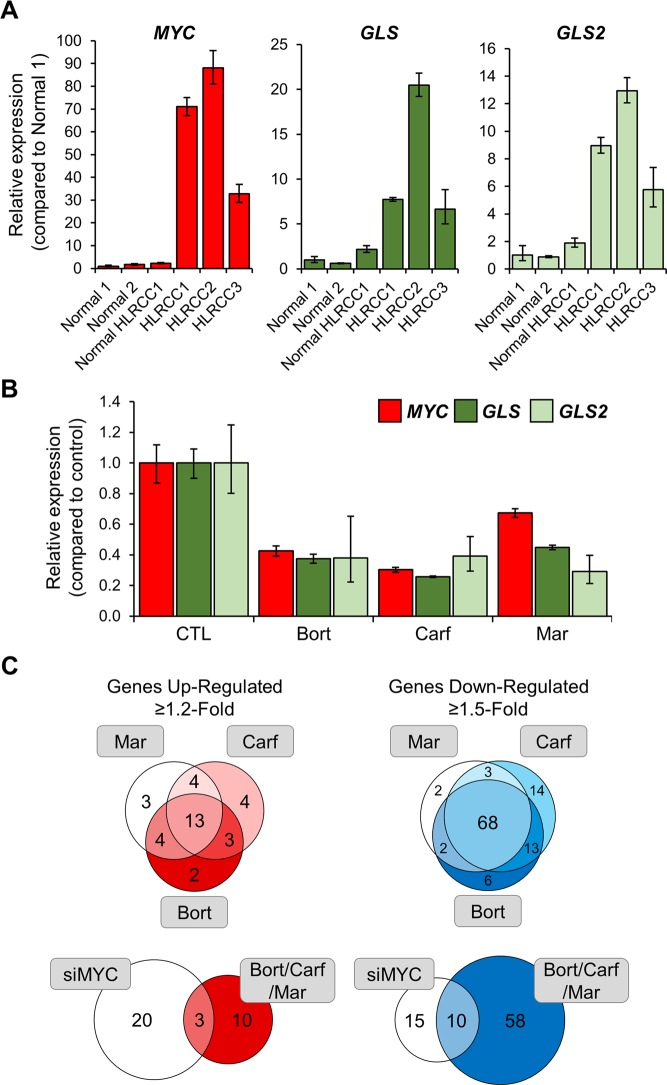
Proteasome inhibition alters glutamine metabolism. (**A**) Relative mRNA expression levels of *MYC*, *GLS* and *GLS2* in a panel of HLRCC specimens; (**B**) Relative mRNA expression levels of *MYC*, *GLS* and *GLS2* in UOK262 after 24 hours treatment with bortezomib, marizomib and carfilzomib (30 nM); (**C**) Venn diagrams of the effects of the proteasome inhibitors and siMYC measured by Nanostring-based mRNA expression of 180 metabolic genes. Bort: bortezomib; Mar: marizomib; Carf: carfilzomib; CTL: DMSO control.

*MYC* is significantly overexpressed in HLRCC-associated FH-deficient tumors compared to associated normal kidney tissue (Fig. 5A). *GLS* and *GLS2* encode the kidney and liver isoforms of the glutaminase enzyme that support glutamine metabolism and the maintenance of the redox balance. *GLS* and *GLS2* are respectively indirect and direct downstream targets of c-Myc^35^ and were significantly overexpressed in FH-deficient tumor specimens (Fig. 5A). The three proteasome inhibitors which downregulated *MYC* expression in HLRCC tumor cells (Fig. 4C), also decreased the expression of *GLS* and *GLS2* (Fig. 5B). This indicates that glutamine metabolism may also be modulated by proteasome inhibition.

**Figure 6:**
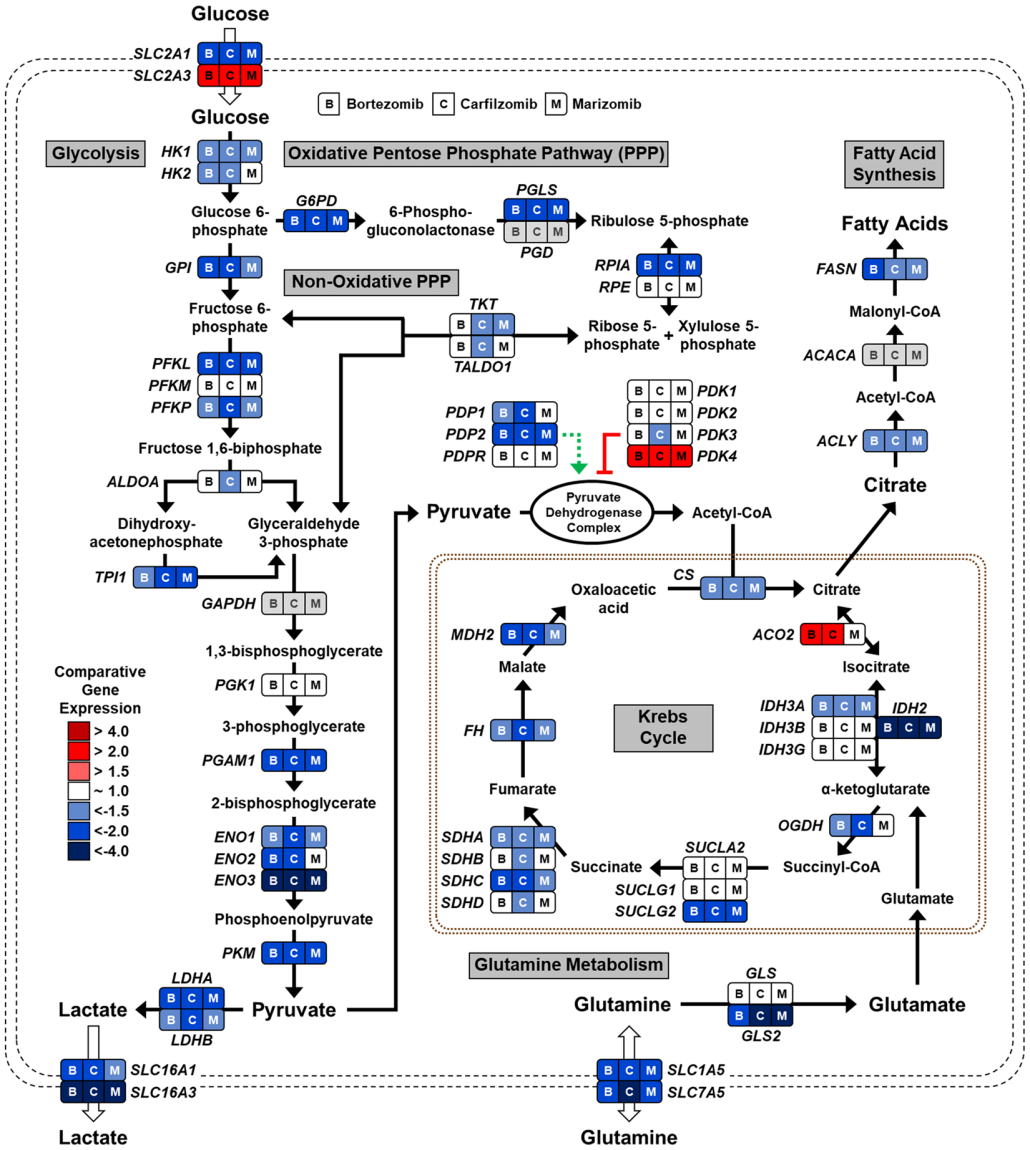
Proteasome inhibition downregulates both glucose and glutamine metabolism. The comparative gene expression of metabolic genes between UOK262 cells treated with each of the three proteasome inhibitors (bortezomib, carfilzomib, or marizomib) and the control DMSO treated UOK262 cells were calculated and represented on a schematic of the major metabolic pathways. B: bortezomib; M: marizomib; C: carfilzomib.

To further evaluate the metabolic processes affected by proteasome inhibition, we performed a targeted metabolism profiling assay. Nanostring-based mRNA expression profiling of 180 metabolic genes and 10 reference genes was used to compare untreated UOK262 cells to proteasome inhibitors treated UOK262 cells, and UOK262 cells transiently silenced with a non-targeting sequence (siCTL) to UOK262 cells transiently silenced with siMYC (Table S1). The proteasome inhibitors had similar profiles with 68 out of the 108 (63%) genes downregulated 1.5-fold and 13 out of 33 (39%) genes upregulated 1.2-fold shared by all three proteasome inhibitors (Fig. 5C). The samples with siMYC silencing presented 3 commonly upregulated genes (1.2- fold) with the proteasome inhibitors, 10 genes commonly downregulated (1.5-fold), and 40 genes commonly downregulated (1.2-fold), that included *GLS2*, *LDHA*, and the glutamine transporters *SLC7A5* and *SLC1A5* (Fig. 5C and Table S1). This supports the concept that c-Myc may partially play a role in mediating the proteasome inhibitors metabolic effect (at least related to LDHA expression), although there are also significant c-Myc-independent proteasome inhibitors induced metabolic effects. Mapping the genes either up- or downregulated 1.5-fold across all three proteasome inhibitor treatments showed a general downregulation of aerobic glycolysis, the oxidative pentose phosphate pathway, and glutamine metabolism, which may affect the cells’ ability to perform glutathione synthesis and to support the redox response capacities (Fig. 6 and Table S1). It is however uncertain whether these events observed *in vitro* after an acute treatment would be translatable to a clinical setting, hence further translational and clinical characterization of the metabolic effects of proteasome inhibitors is required.

## Discussion

HLRCC-associated Type 2 papillary renal cell carcinoma has a propensity to metastasize to local lymph nodes as well as distant sites and there is currently no standard therapy for patients with advanced disease. We have previously shown the cytotoxic sensitivity of HLRCC to the proteasome inhibitor bortezomib^27^. Two proteasome inhibitors, bortezomib and carfilzomib, have been approved by the US Food and Drug Administration for the treatment of multiple myeloma^36^. However, they cannot cross the BBB, unlike the second-generation of proteasome inhibitor marizomib, which is BBB-permeant. Similar to bortezomib, marizomib was found to have a significant cytotoxic effect against the FH-deficient HLRCC cell line model, UOK262. UOK262 was highly sensitive to marizomib *in vitro* with an IC50~5–6 nM and *in vivo* with complete regression of UOK262 xenografts in 80% of mice, while it had very little effect on the FH restored cell line, UOK262WT. As previously shown in other models, the proteasome inhibitors cytotoxic-effects were at least partly ROS-dependent, and their cytotoxicity was partially reversed using the ROS scavenger NAC.

Surprisingly, the three proteasome inhibitors reduced *in vitro* glucose uptake and lactate production of UOK262 cells. This was confirmed *in vivo* by a hyperpolarized ^13^C pyruvate MRI study showing that marizomib significantly inhibited the rate of pyruvate to lactate flux in a mouse xenograft model of HLRCC after only a 48 h treatment. To further understand how the proteasome inhibitors modulated UOK262 metabolism, we evaluated the role of p62 and c-Myc in regulating LDHA expression^33^. The three proteasome inhibitors led to the degradation of p62, which was associated with decreased *MYC* expression. C-Myc is a well-known transcription factor with numerous downstream targets, one of which is *LDHA*. P62-mediated *MYC* decrease led to a decreased *LDHA* mRNA and protein expression. Transient silencing of *SQSTM1* (p62) and *MYC* with pooled small interference RNA further validated this mechanism. Additionally, in HLRCC cells, c-Myc direct and undirect targets *GLS* and *GLS2* were upregulated. Treatment with the three proteasome inhibitors decreased the expression of *GLS* and *GLS2* in UOK262, highlighting a modulation of glutamine metabolism by the proteasome inhibitors, which may impact the cells’ anti-oxidant response capacity^37^. Analysis of the wider expression of genes involved in cellular metabolism further showed a general reduction in expression of genes involved in glycolysis, glutamine metabolism, and fatty acid metabolism. Transient silencing of *MYC* only partially recapitulated the metabolic effects of the proteasome inhibitors suggesting that this global metabolic effect was both c-Myc-dependent and c-Myc-independent.

In this study, we uncovered a metabolic effect of proteasome inhibitors in FH-deficient kidney cancer cells *in vitro* and *in vivo*. Proteasome inhibition disrupted glucose and glutamine metabolism, restricting nutrients and lowering the cells’ anti-oxidant response capacity. Additional work is necessary prior to apply these findings clinically as it is still unclear whether the metabolic effects of the proteasome inhibitors correlate with therapeutic response or could be exploited within a therapeutic strategy. In conclusion, these findings increase our understanding of the mechanism of action of proteasome inhibitors in HLRCC cells and may provide the foundations for the development of markers of efficacy and the development of effective therapeutic strategies for HLRCC tumors, especially brain metastases using a BBB-permeant proteasome inhibitor.

## Methods

### Cell lines and cell culture

UOK262 and UOK262WT were established in the Urologic Oncology Branch from surgically resected tumor specimens (National Cancer Institute, Bethesda, MD)^14,25^. Cells were cultured in high glucose DMEM without pyruvate supplemented with 10% FBS. The cells were harvested or treated when they reached 70–80% confluence.

### Chemical agents

Marizomib was generously provided by Nereus Pharmaceuticals. All other compounds used were from Sigma-Aldrich (St. Louis, MO) or Selleck Chemicals (Houston, TX).

### Cell viability

Cell viability was measured using a Cell-Titer Glo purchased from Promega Biosciences, Inc. (San Luis Obispo, CA), following the manufacturer’s protocol.

### ATP assay

ATP levels were determined 4 h post-treatment using the ATPLite assay (PerkinElmer, Shelton, Connecticut), following the manufacturer’s protocol.

### Immunoblotting

Ten to twenty micrograms of protein were loaded in 4–20% polyacrylamide gels (Biorad, Hercules, CA). After electrophoresis, proteins were transferred to PVDF membranes, blocked with 2.5% fat-free milk for 1 h, and incubated with primary antibodies overnight at 4 °C under gentle rocking. The following day, membranes were washed three times with TBS-Tween, and blots were incubated with horseradish peroxidase-linked secondary antibodies (Sigma-Aldrich) for 1 h before development with the ECL protein detection system (Thermo Fisher Scientific, Rockford, IL). Rabbit antibodies against LDH-A, p62, c-MYC and mouse antibodies against α-tubulin were from Cell Signaling Technology, Inc (Danvers, MA). All antibodies were used freshly diluted at 1:1000.

### ROS assay

Amount of reactive oxygen species in cells after treatment was assessed as previously described^27^. Briefly, 5000 cells were plated in black-well clear bottom 96-well plates (Perkin-Elmer). The following day, cells were pretreated with water or NAC (5 mM) for 4 h and then were treated using proteasome inhibitors or control as indicated in the figures and figure legends for 2 h prior to exposing the cells with 20 µM of cell-permeant 2′,7′- dichlorodihydrofluorescein diacetate (H2DCFDA) for 1 h. Cells were subsequently washed one time with PBS before changing their medium to complete phenol-free DMEM. Ten minutes later, ROS were assessed by measuring fluorescein using the EnSpire plate reader (Perkin Elmer).

### Animal study

Animal experiments were performed in strict accordance with the guidelines of the Animal Care and Use Committee of the National Institutes of Health. The protocol was approved by the NCI Animal Care and Use Committee (Protocol Number: PB-029). Sixteen female athymic nude mice (Taconic, Germantown, NY) were injected on the right flank with 4 million UOK262 cells diluted in matrigel (100%; BD Bioscience, San Jose, CA). Tumors were measured weekly using calipers and tumor volume was calculated as length × width^2^. Four weeks after injection, tumor volume reached 100 mm^3^. Mice were randomized into two groups of eight mice each. One group was treated with marizomib (150 µg/kg twice a week by intraperitoneal (i.p.) injection) and the second group was treated vehicle (PBS/DMSO; v/v) following an identical schedule. When tumors from the vehicle-treated mice reached 1.5 cm^3^, animals were euthanized by trained personnel using carbon dioxide inhalation following the AVMA Guidelines for the Euthanasia of Animals. For the repeat experiment, twenty mice were randomized into two groups of ten mice each.

### Metabolic analysis of the cells

The metabolism of the cells and their metabolites consumption were assessed by measuring their Extra Cellular Acidification Rate (ECAR) and using a biochemical analyzer. ECAR were measured using the XF96 Extracellular Flux Analyzer from Seahorse Bioscience (Chicopee, MA) using XF96 microplates as previously described^14^. Briefly, cells were seeded at 15,000 per well. Twenty-four hours later, culture media was removed from the XF culture plates, wells were washed one time and cells were incubated with bicarbonate-free DMEM supplemented with glucose (4.5 g/L) and sodium pyruvate (2 mM) (pH 7.4 at 37 °C), with or without treatment as described in the figure legends. Metabolite consumption was calculated using the biochemistry analyzer (YSI technology) to measure the levels of glucose and lactate in the media of the cells before and after 24 h treatment.

### ^13^C MRI of hyperpolarized ^13^C-labeled pyruvate metabolism

Imaging was performed as previously described^28^. Briefly, [1-^13^C] pyruvic acid was polarized at 3.35 T and 1.4 K in a Hypersense DNP Polarizer (Oxford Instruments). After 40–60 min, the hyperpolarized sample was dissolved in 4.5 mL of a superheated alkaline buffer [40 mM 4-(2-hydroxyethyl)-1- piperazineethanesulfonic acid (HEPES), 30 mM NaCl and 100 mg/L ethylendiaminetetraacetic acid (EDTA)]. pH was adjusted to 7.4 using NaOH after mixture with [1-^13^C] pyruvic acid. Hyperpolarized [1-^13^C] pyruvate solution (93 mmol/L, 12 µL/g body weight) was intravenously injected through a catheter placed in the tail vein of the mouse. Hyperpolarized ^13^C MRI studies were performed on a 7 T scanner (Bruker Bio- Spin MRI GmbH) using a 17 mm home-built ^13^C solenoid coil placed inside of a saddle coil for 1 H. The ^13^C two-dimensional spectroscopic images were acquired 30 seconds after the start of pyruvate injection from a 28 × 28 mm field of view in an 8 mm coronal slice through the tumor, with matrix size of 16 × 16, spectral width of 8 kHz, repetition time (TR) 78 ms, 0.2 ms Gaussian excitation pulse with a flip angle of 10°. The total time required to acquire an image was 20 seconds.

### Flow cytometry

Autophagy was detected by flow cytometry using a green fluorescent cationic amphiphilic tracer dye that labels phagophores, autophagosomes, and autolysosomes, and minimally stains lysosomes (#ab139484, Abcam) as per the manufacturer protocol.

### RNA extraction and RT-PCR analysis

Total RNA was extracted from frozen tumors, normal tissues or cell lines using Trizol Reagent (Invitrogen, Carlsbad, CA, USA) as previously described^28^. For RT-PCR, cDNA was reverse-transcribed from 2 µg of total mRNA to cDNA using the SuperScript® VILO™ cDNA Synthesis Kit (Invitrogen, Carlsbad, CA, USA) in a 20 µL volume. The cDNAs were further diluted with 70 µL of RNase-Free Water and 2 µL was used in 10 µL reaction volume for RT-PCR amplification using an ABI ViiA7 real-time PCR system (Applied Biosystems). Expression levels were normalized to the control housekeeping gene ACTB (Hs99999903_m1). The primers and fluorogenic probe Taqman® Gene Expression Assays used are: LDHA (Hs01378790_g1), SQSTM1 (Hs01061917_g1), MYC (Hs00153408_m1), GLS (Hs01014020_m1), GLS2 (Hs00998733_m1).

### nCounter vantage™ RNA cancer metabolism panel (nanostring technology)

RNA extraction from treated and untreated UOK262 cells was performed using the RNeasy kit (QIAGEN). The Cancer Metabolism Panel commercial array was performed using 50 ng of RNA and was analyzed as recommended by the manufacturer. The array includes 12 housekeeping genes and 180 genes related to cancer metabolism. Data for 10 out of the 12 housekeeping genes provided consistent comparative expression values and this was used to normalize between samples.

### Statistics

All values are expressed as mean ± standard error. All experiments have been performed three times, with exception of the animal study which was performed two times. Values were compared using the Student-Newman-Keul’s test. P < 0.05 was considered significant.

## Supplementary information

Supplementary Information

## Data Availability

All data generated or analyzed during this study are included in this published article (and its Supplementary Information Files).
