# Supplementary material for: Proteasome inhibition disrupts the metabolism of fumarate hydratase- deficient tumors by downregulating p62 and c-Myc
Source: Sci Rep. 2019 Dec 5;9:18409. doi: 10.1038/s41598-019-55003-2 (PMC6895110; doi:10.1038/s41598-019-55003-2)
Supplement: Supplementary file 1 — Supplementary Information [file 41598_2019_55003_MOESM1_ESM.pdf]

Supplementary information

Proteasome inhibition disrupts the metabolism of fumarate hydratase- deficient tumors by downregulating p62 and c-Myc.

Carole Sourbier[1,2*], Christopher J. Ricketts[1], Pei-Jyun Liao[1,2], Shingo Matsumoto[3,4,5], Darmood Wei[1], Martin Lang[1], Reema Railkar[1], Youfeng Yang[1], Ming-Hui Wei[1], Piyush Agarwal[1], Murali Krishna[3], James B. Mitchell[3], Jane B. Trepel[6], Len Neckers[1] and W. Marston Linehan[1*]

Figure S1. Time-dependent effect of marizomib on UOK262 cells viability.

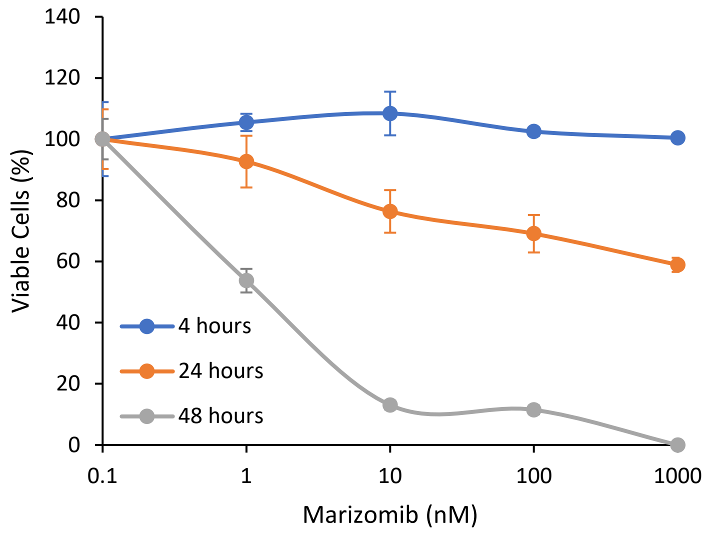

UOK262 cells were treated for 4 hours, 24 hours or 48 hours with a concentration range of marizomib (from 0.1 nM to 1µM). Cell viability was assessed by CellTiterGlo.

Figure S2. Original full size and unprocessed blots included in Figure 3A.

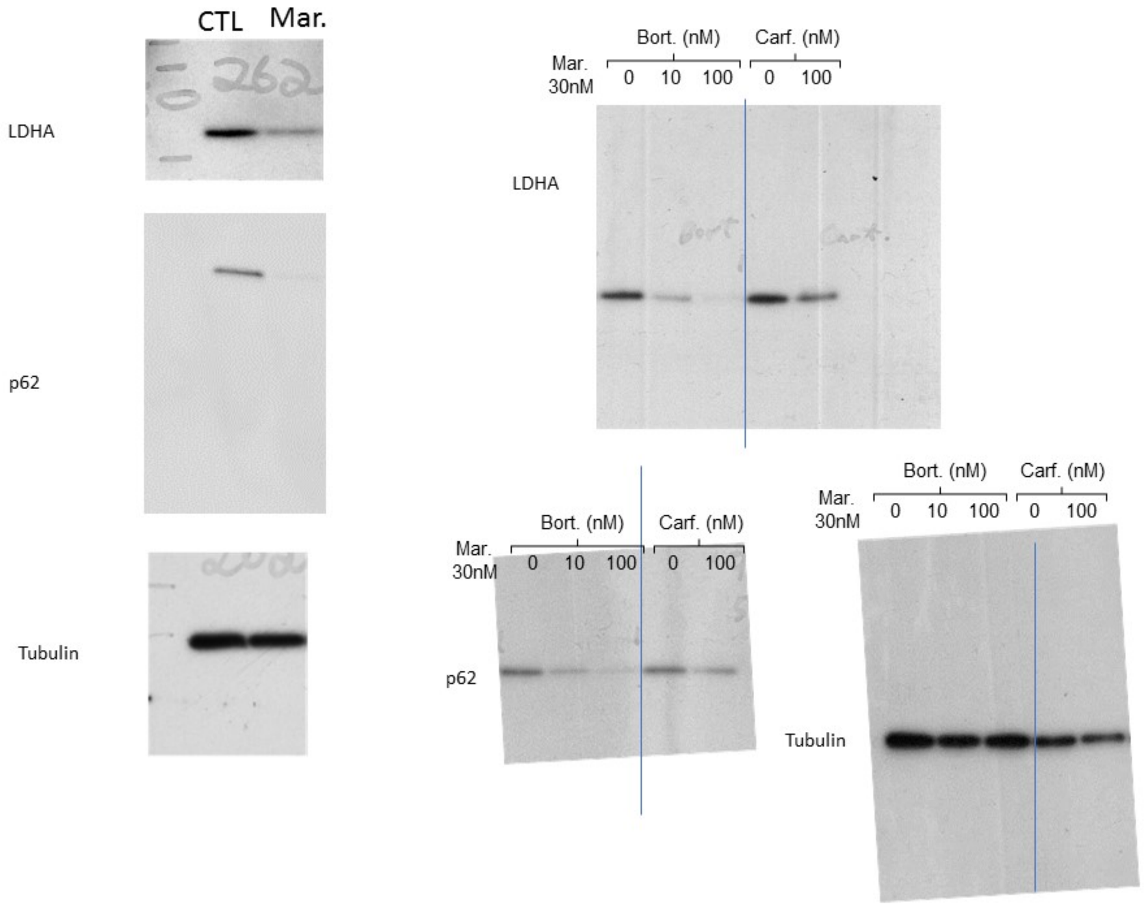

Figure S3. Effect of Proteasome inhibitors on SQSTM1 mRNA expression levels and c-Myc protein expression levels in UOK262 cells treated with proteasome inhibitors.

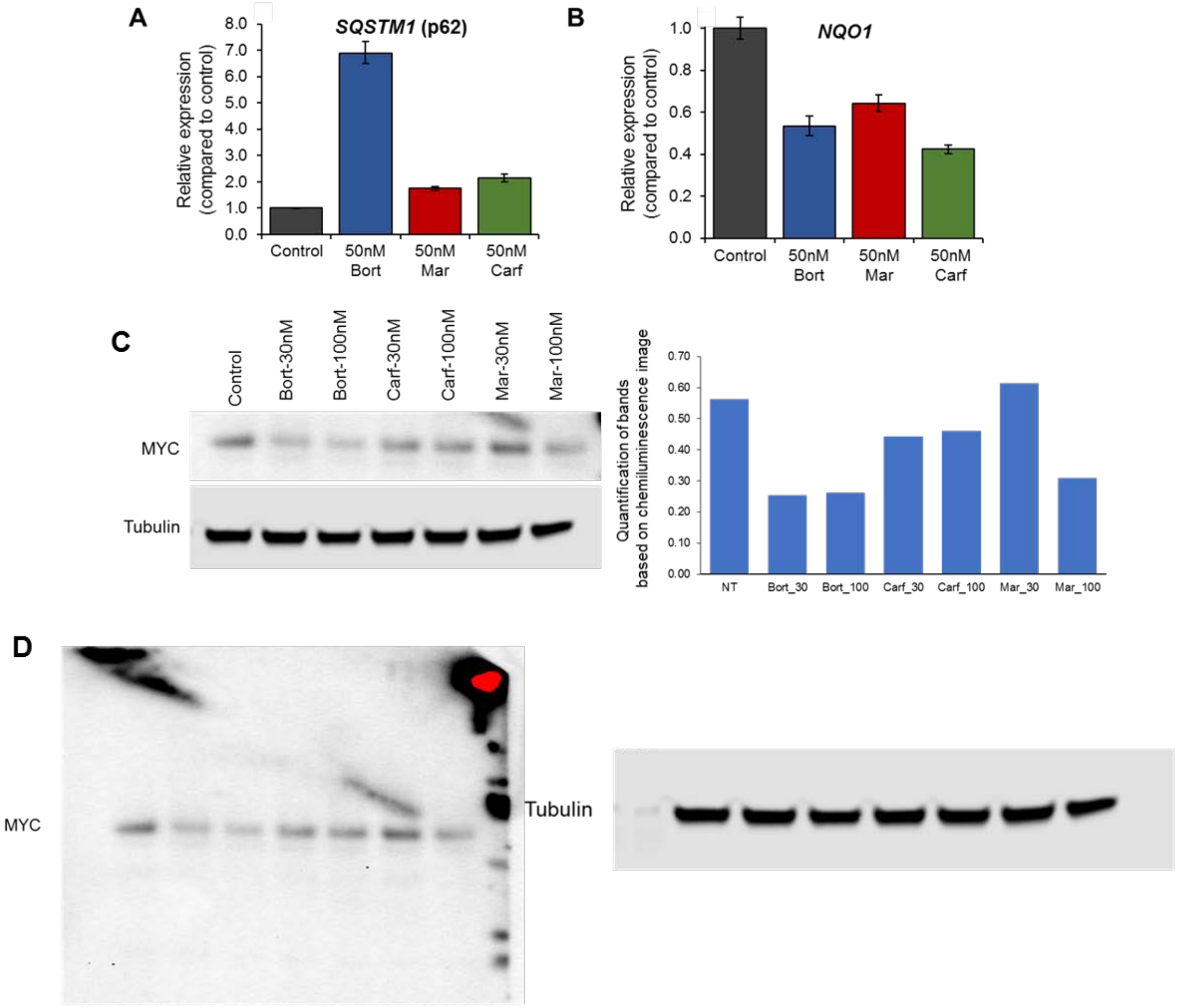

A-B: Effect of Proteasome inhibitors on SQSTM1 and NQO1 mRNA expression levels; C: c-Myc protein expression after proteasome inhibitors' treatment; D: Original full size and unprocessed blots related to Figure S3C.

Table S1

Gene	Bort	Carf	Mariz	siMYC
NOXO1	0.6018	0.4103	0.1518	2.0393
IDH2	0.1261	0.0992	0.1596	0.8434
H6PD	0.0798	0.0793	0.2013	0.6329
ENO3	0.1511	0.1376	0.2101	1.1224
GLS2	0.2686	0.1224	0.2217	0.4196
CAD	0.1806	0.1630	0.2352	0.6777
SLC16A3	0.1674	0.1714	0.2389	0.9424
SLC7A5	0.2525	0.1936	0.2989	0.7729
SHMT1	0.2444	0.2383	0.3087	0.7081
NOXA1	0.0812	0.1473	0.3203	1.3065
LDHA	0.3092	0.2988	0.3305	0.7340
TP53	0.2807	0.2131	0.3430	0.6344
RPIA	0.2867	0.2510	0.3634	0.6150
PDGFC	0.2248	0.2543	0.3705	1.2540
SLC5A2	0.3348	0.4354	0.3790	0.5650
NTRK1	0.1559	0.2720	0.3860	0.8579
PKM	0.3745	0.3305	0.3901	0.9439
PRPS2	0.3194	0.2898	0.3978	0.8416
HIF1A	0.3901	0.3715	0.4026	1.1070
SLC1A5	0.3837	0.3382	0.4036	0.7448
IKBKB	0.3064	0.2958	0.4056	1.0449
FGFR2	0.2343	0.2181	0.4076	1.0250
SLC25A1	0.3376	0.3351	0.4135	0.7348
AKT1	0.3515	0.3612	0.4207	0.9746
PFKL	0.3674	0.3643	0.4228	0.8488
EIF4EBP1	0.3430	0.3021	0.4252	0.6340
SUCLG2	0.4065	0.4002	0.4308	0.8458
PGLS	0.3584	0.3258	0.4374	0.7983
TPI1	0.5015	0.4149	0.4480	0.7953
G6PD	0.4678	0.4111	0.4561	0.8154
VEGFA	0.4000	0.4486	0.4592	1.0763
AKT2	0.4476	0.4121	0.4760	0.8608
VHL	0.3676	0.4053	0.4772	1.0352
SLC2A1	0.4735	0.3416	0.4786	0.9825
HRAS	0.5087	0.3818	0.4904	0.9040
PDP2	0.3891	0.4284	0.4914	0.8449
SP1	0.5692	0.5689	0.4965	0.9152
PGAM1	0.4269	0.3634	0.4989	0.9004
RAC2	0.3819	0.3451	0.5171	1.0528

LDHB	0.5126	0.4442	0.5178	0.5795
GPI	0.4958	0.4484	0.5192	0.7861
SCO2	0.4603	0.4970	0.5276	0.7673
ERBB2	0.4856	0.5483	0.5288	0.9454
ENO1	0.5803	0.4719	0.5344	0.8060
MLST8	0.4566	0.4692	0.5373	0.6451
FH	0.5327	0.4942	0.5426	0.8311
TIGAR	0.4593	0.6198	0.5450	0.9867
MDH2	0.4939	0.3879	0.5517	0.6202
RAC1	0.4937	0.4752	0.5530	0.9066
FASN	0.4100	0.5056	0.5570	0.4630
CS	0.5375	0.5110	0.5741	0.8318
SDHC	0.4708	0.4288	0.5881	1.0345
SDHA	0.6599	0.6382	0.6018	1.0291
ACLY	0.5879	0.5911	0.6036	0.9266
PRKAA2	0.4510	0.4505	0.6065	0.8511
SLC44A4	0.3945	0.4789	0.6081	1.1423
RPS6KB2	0.5470	0.4619	0.6107	0.7805
PDGFA	0.4245	0.4293	0.6123	1.1475
SLC16A1	0.4308	0.4714	0.6126	0.6950
MDH1B	0.2254	0.3651	0.6204	0.7255
ACO1	0.5691	0.5834	0.6227	0.8218
EGLN1	0.5833	0.6459	0.6241	0.9474
PFKP	0.5636	0.4370	0.6250	1.1633
PRKAA1	0.5985	0.6385	0.6271	0.9257
EGFR	0.4784	0.4478	0.6281	1.3685
HK1	0.5632	0.5522	0.6332	0.9227
IDH3A	0.6008	0.5730	0.6453	0.8934
PIK3CB	0.4713	0.5741	0.6473	0.9694
SLC2A4	1.0520	0.4781	0.2653	1.6331
HK3	0.8606	0.2610	0.4741	1.0611
TKT	0.6697	0.6645	0.5782	0.6944
FBP1	0.5844	0.7459	0.5798	2.1222
ARNT	0.5646	0.6984	0.6363	0.7946
PRKCB	0.4360	0.4219	0.7020	0.8161
MET	0.5480	0.5947	0.7118	1.1045
PDP1	0.5042	0.4732	0.7146	1.0714
AKT3	0.6411	0.6028	0.7243	0.9328
OGDH	0.5235	0.4686	0.7593	0.9703
PDHA1	0.6172	0.5525	0.7673	0.8079
ALDOB	0.2627	0.3590	0.7824	0.4891

ALDOC	0.4606	0.5351	0.8094	0.5744
FGFR3	0.2564	0.5607	0.8135	1.0492
ENO2	0.4924	0.4764	0.8543	1.6690
PLD1	0.5428	0.6490	0.8825	1.4493
EGLN3	0.6020	0.6321	0.8927	1.1080
HK2	0.5237	0.5397	0.9723	0.6298
G6PC	0.9019	0.9587	0.1518	1.6331
NOX1	1.2020	0.8220	0.5950	0.5444
ALDOA	0.7301	0.5731	0.6673	0.8576
IDH1	0.5496	0.6670	0.6693	0.9291
RBKS	0.6680	0.5805	0.6854	0.9327
KRAS	0.6510	0.7619	0.6900	1.0387
PDHB	0.6971	0.6340	0.6949	0.8739
DLAT	0.7233	0.6561	0.7108	0.8629
SDHB	0.6803	0.5526	0.7121	0.7289
MDH1	0.6902	0.5588	0.7483	0.8105
SDHD	0.7139	0.5954	0.7492	0.7283
TALDO1	0.7407	0.6066	0.7519	1.0107
PDK3	0.7032	0.5866	0.7553	0.7890
PTEN	0.6638	0.7292	0.7674	1.1388
GCK	0.7284	0.5162	0.8017	1.8777
PIK3CD	0.7108	0.6116	0.8603	1.3455
FLT3	0.5260	1.1182	0.8692	1.6331
MYC	0.7151	0.5479	0.9231	0.3267
KIT	0.4509	1.3690	1.0427	0.5939
PDGFRB	0.6660	0.7025	1.1465	0.5529
PRKCG	1.4061	0.3329	1.3902	0.5444
RET	0.8761	0.6380	2.0837	0.4666
PGAM2	1.4061	1.5977	0.6951	1.0887
PFKM	0.8182	0.8695	0.7003	0.5885
G6PC3	0.7841	0.7228	0.7016	0.7556
SIRT3	0.7808	0.8111	0.7225	0.7735
MAPK3	0.7932	0.7847	0.7245	0.9266
EIF2B4	0.7889	0.8218	0.7362	0.9305
PDHA2	0.7511	1.1638	0.7445	1.8777
EGLN2	0.8596	0.8431	0.7660	1.0078
PPARGC1A	0.7039	0.7307	0.7786	1.1855
OAZ2	0.8382	0.7182	0.7812	1.0494
EIF4E2	0.7360	0.6904	0.7890	0.8952
DLST	0.7346	0.7186	0.7913	0.9357
GLS	0.8546	0.7979	0.7923	1.3282

**PLD2**	0.7488	0.8439	0.8110	0.9772
**MTOR**	0.6713	0.7546	0.8232	0.9196
**MAPK1**	0.8151	0.8521	0.8339	1.0621
**DLD**	0.8490	0.7781	0.8477	0.9176
**RPE**	0.7882	0.7219	0.8519	0.9014
**BECN1**	0.7155	0.6898	0.8529	1.0351
**PLCG1**	0.8547	0.9600	0.8597	1.0749
**FGFR1**	0.8140	0.7763	0.8599	1.1970
**RPS6KB1**	0.9232	1.0449	0.8658	1.0001
**GSK3A**	0.9576	0.9174	0.8674	0.9517
**SLC7A11**	0.6996	0.8237	0.8728	1.2543
**SIRT6**	0.7859	0.6838	0.8795	0.7538
**NTRK3**	1.0523	1.2778	0.9032	0.7140
**SLC38A2**	0.8403	0.9776	0.9082	0.8463
**SUCLG1**	1.0068	0.8312	0.9229	0.7549
**GYS1**	0.8829	0.9270	0.9262	1.2001
**RAF1**	0.9211	0.9273	0.9381	0.9749
**NRAS**	0.7614	0.6848	0.9486	1.2018
**GALM**	0.8043	1.1356	0.9533	0.8497
**MAPKAP1**	0.8219	0.7109	0.9689	0.8261
**PRKAB1**	1.1162	1.1262	0.9741	1.0596
**MAP2K2**	1.1315	1.0114	0.9854	0.8335
**SUCLA2**	1.0728	1.0712	0.9946	0.9806
**PDPR**	0.7874	0.8717	0.9995	0.9913
**PDK1**	0.7424	0.8172	1.0166	0.7648
**HKDC1**	1.0898	1.1930	1.0328	1.1393
**NOX4**	0.7883	1.6760	1.0401	0.9799
**NOX3**	1.4061	0.9586	1.0427	2.4454
**GBE1**	0.8428	0.9675	1.0635	0.9200
**RICTOR**	1.0653	1.1556	1.0660	0.9025
**PIK3CA**	1.0768	1.2715	1.0999	1.1296
**PGK1**	1.0541	0.8840	1.1152	0.8076
**IDH3G**	1.1055	0.9466	1.1181	0.9678
**PRPS1**	1.1799	1.1010	1.1513	1.0582
**TSC2**	1.2514	1.2378	1.1677	0.9993
**FOXO3**	1.2091	1.2294	1.1833	1.1683
**EPAS1**	1.0332	1.0068	1.1860	0.9189
**IDH3B**	1.2298	1.1294	1.2036	0.8675
**RPTOR**	1.0899	1.0730	1.2103	0.9440
**TSC1**	1.0829	1.2417	1.2188	0.9181
**PDK2**	1.0965	1.2978	1.2418	0.7895

**ACO2**	1.6728	1.5719	1.3782	0.9824
**MAP2K1**	1.3642	1.2872	1.3815	1.0416
**PRKAB2**	1.8016	1.9582	1.3873	1.0751
**PDGFRA**	0.8190	1.2779	1.3899	0.5650
**BPGM**	1.0770	1.4113	1.4416	0.8266
**EGF**	1.6562	1.5774	1.5259	1.3303
**NOX5**	1.0972	1.1247	1.6311	1.3748
**PDK4**	1.5023	1.7517	1.6673	0.5529
**JUN**	2.0311	1.6925	1.8357	1.3442
**OAZ3**	1.3612	1.1273	1.9314	1.2961
**ODC1**	1.3162	0.8774	2.0661	0.6541
**EIF2AK3**	1.5463	2.1477	2.2397	1.3677
**SLC2A3**	2.4703	2.2043	2.7858	0.8819
**TKTL2**	3.1611	2.8759	3.1280	0.8151
**SLC5A1**	2.1091	3.3602	3.1333	1.1423
**SLC2A2**	2.4597	2.5597	3.4791	0.4666
**PKLR**	2.6325	2.8809	3.6547	0.4079
**ROS1**	2.1051	2.8761	4.4023	0.9975

>1.2 UP	>1.5 UP	>1.2 DOWN	>1.5 DOWN
0	0	3	3
0	0	3	3
0	0	3	3
0	0	3	3
0	0	3	3
0	0	3	3
0	0	3	3
0	0	3	3
0	0	3	3
0	0	3	3
0	0	3	3
0	0	3	3
0	0	3	3
0	0	3	3
0	0	3	3
0	0	3	3
0	0	3	3
0	0	3	3
0	0	3	3
0	0	3	3
0	0	3	3
0	0	3	3
0	0	3	3
0	0	3	3
0	0	3	3
0	0	3	3
0	0	3	3
0	0	3	3
0	0	3	3
0	0	3	3
0	0	3	3
0	0	3	3
0	0	3	3
0	0	3	3
0	0	3	3
0	0	3	3
0	0	3	3
0	0	3	3
0	0	3	3

0	0	3	3
0	0	3	3
0	0	3	3
0	0	3	3
0	0	3	3
0	0	3	3
0	0	3	3
0	0	3	3
0	0	3	3
0	0	3	3
0	0	3	3
0	0	3	3
0	0	3	3
0	0	3	3
0	0	3	3
0	0	3	3
0	0	3	3
0	0	3	3
0	0	3	3
0	0	3	3
0	0	3	3
0	0	3	3
0	0	3	3
0	0	3	3
0	0	3	3
0	0	3	3
0	0	3	3
0	0	3	3
0	0	3	3
0	0	2	2
0	0	2	2
0	0	3	2
0	0	3	2
0	0	3	2
0	0	3	2
0	0	3	2
0	0	3	2
0	0	3	2
0	0	3	2
0	0	3	2
0	0	3	2

0	0	3	2
0	0	3	2
0	0	2	2
0	0	2	2
0	0	2	2
0	0	2	2
0	0	1	1
1	0	2	1
0	0	3	1
0	0	3	1
0	0	3	1
0	0	3	1
0	0	3	1
0	0	3	1
0	0	3	1
0	0	3	1
0	0	3	1
0	0	3	1
0	0	3	1
0	0	3	1
0	0	3	1
0	0	2	1
0	0	1	1
0	0	2	1
1	0	1	1
0	0	2	1
2	0	1	1
1	1	1	1
2	1	1	0
0	0	2	0
0	0	3	0
0	0	3	0
0	0	3	0
0	0	3	0
0	0	2	0
0	0	1	0
0	0	3	0
0	0	2	0
0	0	3	0
0	0	3	0
0	0	2	0

0	0	2	0
0	0	3	0
0	0	1	0
0	0	1	0
0	0	2	0
0	0	2	0
0	0	0	0
0	0	2	0
0	0	0	0
0	0	0	0
0	0	2	0
0	0	2	0
1	0	0	0
0	0	0	0
0	0	1	0
0	0	0	0
0	0	0	0
0	0	2	0
0	0	1	0
0	0	2	0
0	0	0	0
0	0	0	0
0	0	0	0
0	0	1	0
0	0	2	0
0	0	0	0
1	1	1	0
1	0	0	0
0	0	0	0
0	0	0	0
1	0	0	0
0	0	0	0
0	0	0	0
0	0	0	0
2	0	0	0
2	0	0	0
0	0	0	0
2	0	0	0
1	0	0	0
2	0	0	0
2	0	0	0

3	2	0	0
3	0	0	0
3	2	0	0
2	0	1	0
2	0	0	0
3	3	0	0
1	1	0	0
3	3	0	0
3	3	0	0
2	1	0	0
2	1	0	0
3	3	0	0
3	3	0	0
3	3	0	0
3	3	0	0
3	3	0	0
3	3	0	0
3	3	0	0
